# A Health eLearning Ontology and Procedural Reasoning Approach for Developing Personalized Courses to Teach Patients about Their Medical Condition and Treatment

**DOI:** 10.3390/ijerph18147355

**Published:** 2021-07-09

**Authors:** Martin Michalowski, Szymon Wilk, Wojtek Michalowski, Dympna O’Sullivan, Silvia Bonaccio, Enea Parimbelli, Marc Carrier, Grégoire Le Gal, Stephen Kingwell, Mor Peleg

**Affiliations:** 1 Nursing Informatics, School of Nursing, University of Minnesota, Minneapolis, MN 55455, USA; 2 Institute of Computing Science, Poznan University of Technology, 60-965 Poznań, Poland; szymon.wilk@cs.put.poznan.pl; 3 Telfer School of Management, University of Ottawa, Ottawa, ON K1N 6N5, Canada; wojtek@telfer.uottawa.ca (W.M.); bonaccio@telfer.uottawa.ca (S.B.); 4 School of Computer Science, Technological University Dublin, D02 HW71 Dublin, Ireland; Dympna.OSullivan@tudublin.ie; 5 Department of Electrical, Computer and Biomedical Engineering, University of Pavia, 27100 Pavia, Italy; enea.parimbelli@gmail.com; 6 Division of Hematology, The Ottawa Hospital, Ottawa, ON K1Y 4E9, Canada; mcarrier@toh.ca; 7 Department of Medicine, The Ottawa Hospital, Ottawa, ON K1Y 4E9, Canada; glegal@toh.ca; 8 Department of Orthopaedic Surgery, The Ottawa Hospital, Ottawa, ON K1Y 4E9, Canada; skingwell@toh.ca; 9 Department of Information Systems, University of Haifa, Haifa 3498838, Israel; peleg.mor@gmail.com

**Keywords:** patient education, educational learning, VARK, Bloom’s taxonomy, personalization, ontology, procedural reasoning system, precompiled planning

## Abstract

We propose a methodological framework to support the development of personalized courses that improve patients’ understanding of their condition and prescribed treatment. Inspired by Intelligent Tutoring Systems (ITSs), the framework uses an eLearning ontology to express domain and learner models and to create a course. We combine the ontology with a procedural reasoning approach and precompiled plans to operationalize a design across disease conditions. The resulting courses generated by the framework are personalized across four patient axes—condition and treatment, comprehension level, learning style based on the VARK (Visual, Aural, Read/write, Kinesthetic) presentation model, and the level of understanding of specific course content according to Bloom’s taxonomy. Customizing educational materials along these learning axes stimulates and sustains patients’ attention when learning about their conditions or treatment options. Our proposed framework creates a personalized course that prepares patients for their meetings with specialists and educates them about their prescribed treatment. We posit that the improvement in patients’ understanding of prescribed care will result in better outcomes and we validate that the constructs of our framework are appropriate for representing content and deriving personalized courses for two use cases: anticoagulation treatment of an atrial fibrillation patient and lower back pain management to treat a lumbar degenerative disc condition. We conduct a mostly qualitative study supported by a quantitative questionnaire to investigate the acceptability of the framework among the target patient population and medical practitioners.

## 1. Introduction

The adherence of patients to their prescribed therapy is a key factor in successful management of their disease. Non-adherence or poor adherence leads to the worsening of a patient’s condition and ultimately to increased healthcare cost. The review of Devine et al. found that the barriers to adherence (amongst others) include having limited knowledge and understanding of treatment and its side effects combined with low motivation [1]. Jin et al. concluded that additional factors affecting patients’ adherence include the comprehension level of their health care, smoking or alcohol intake, cognitive abilities, and compliance history, as well as treatment-related factors, such as medication administration type, treatment complexity and duration, adverse events, and required degree of behavior modification [2]. Many of the identified barriers are education-based, yet significant advances in educating patients about their disease and treatment are lacking. While patient counseling is commonly used to provide treatment-related information to improve adherence, its scope varies and not all physicians carry it out even though care providers believe that patient education is an important intervention facilitating treatment adherence across a range of conditions and disease severities [3,4,5,6,7]. An intervention involving educational materials customized to patients’ stage of readiness to change, amongst other factors, is an important aspect impacting patients’ attitudes and, consequently, improving their treatment adherence [8].

In previous work [9], we focused on improving patients’ motivation and competence via a mobile application relying on the trans-theoretical model of behavior change [10] and specific behavioral change techniques [11]. However, we did not consider customized educational materials, as postulated by the physicians. Extending our work, we propose a framework to support personalized educational interventions to improve patient understanding of their care. Evidence shows that tailoring communication to a patient’s level of comprehension of their health care is a means to impact adherence to prescribed treatment [12].

We present a framework that develops and delivers educational materials in the form of multi-modal courses, customized to a patient in a manner suitable for the outpatient setting. Inspired by Intelligent Tutoring Systems (ITSs) [13], it uses interactive sessions of dynamically-generated question answering and content delivery to increase a patient’s level of understanding based on the VARK (Visual, Aural, Read/write, Kinesthetic) presentation model [14] and Bloom’s taxonomy of educational objectives [15]. We formalize our framework in two clinical use cases—anticoagulation treatment of an atrial fibrillation patient and lower back pain management for a patient with lumbar degenerative disc condition. Following user-centered design of information system principles [16], together with the stakeholders in our research team (three clinical specialists and six patient representatives), we develop a proof-of-concept implementation to test if the framework can generate courses from its knowledge repository, and if patients find the developed courses useful, and an improvement over existing static educational interventions. We conduct a mostly qualitative study supported by a quantitative questionnaire to investigate the acceptability of the generated courses among the patient target population and medical practitioners.

## 2. Background

An ontology is a specification of a conceptualization [17,18]. It is a description of the concepts and relationships that can exist for an agent or a community of agents. Ontologies enable knowledge sharing and reuse and serve as an agreement to use a vocabulary (i.e., ask queries and make assertions) in a way that is consistent (but not complete) with respect to the specified theory [19]. The use of an ontology facilities the capture and use of knowledge needed to create courses for the breadth of diseases and learning styles supported by our proposed framework.

Typically, educational materials provided to patients include information about conditions and treatments and aim to equip them with knowledge and skills required to self-manage their condition and make informed decisions about subsequent treatment and daily care [20]. Patient education materials are most commonly available as printed pamphlets or references to hospital and professional medical societies websites. This generic standardized information is available to all patients regardless of their comprehension level of their health care. A recent Cochrane review on the effectiveness of printed educational materials found they may have a small beneficial effect on professional practice outcomes, but that there is insufficient information to reliably estimate their effect on patient outcomes [21].

Increasingly, technological solutions are used to deliver patient education materials [22]. Recent years have seen a large increase in the number of medical applications (apps) or e-learning systems with the aim of teaching patients about diseases, drugs, medical tests, and treatments. For example, the MedlinePlus app (U.S. National Library of Medicine, Bethesda, MD, USA) developed by the National Library of Medicine, provides information about diseases, conditions, and wellness (https://medlineplus.gov/, accessed on 7 July 2021). Similar to printed educational materials, the information provided by the app is generic and standardized. Short Message Service (SMS), the most common form of technology-based intervention to deliver tailored content, is used, for example, in asthma [23], diabetes [24] and cardiac care education [25]. Tailored health communication motivates patients to process health messages and improve their health behaviors [26].

Although SMS provides some customization over generic pamphlets, the personalization of content permitted by these messages is limited. We are interested in developing a comprehensive solution for personalized patient education and are inspired by research on ITSs which use Artificial Intelligence to model an explicit encoding of domain knowledge and pedagogic expertise [13]. ITSs have been widely used in education and corporate training and in supporting clinicians’ education [27,28,29]. Thus far, ITSs have very limited application in patient education for health behavior change, with one exception being the Genetic Breast Cancer Risk ITS [30], which uses Fuzzy-Trace Theory to help women understand and make decisions about genetic testing for breast cancer risk.

Researchers have attempted to develop tools for creating personalized patient education materials using ontological approaches implementing various ITS aspects. For example, Chammas et al. [31] proposed a computational tool composed of an ontology and semantic rules for diabetic patients that provides advice for mitigating diabetic complications. The ontology captures patient information such as podiatry observations, symptoms, lifestyle factors, and medical test results. Semantic rules determine the category of guidance and advice provided to a patient. Adnan et al. [32] developed a medication information ontology, which models the medication knowledge necessary for patients to manage their post-discharge self-care. A semantic annotation engine using the GATE (General Architecture of Text Engineering) natural language processer relies on patient details, discharge medications, and ontology of medical information to generate personalized medical advice.

Additionally, Amith et al. [33] developed the Vaccine Information Statement Ontology (VISO) using the Web Ontology Language (OWL) to mitigate the knowledge gap that often exists between patients seeking accurate and reliable information about vaccines and complex or inaccurate sources. Abidi et al. [34] developed a self-management framework for chronic cardiac conditions that uses Social Cognition Theory to provide educational content and strategies, assessment tools and the personalization logic modeled using an OWL-DL-based ontology. The execution of the knowledge encapsulated within the ontology allows for the dynamic generation of a patient’s profile and the selection of the relevant self-management strategies, educational, and motivational messages. Quinn et al. [35] developed an approach using an ontology, a set of rules, and a repository of educational materials for personalization of diabetes treatment. Their ontology models information relating to four main entities (patient, medical conditions, physical activities, and educational content). Bickmore et al. [36] developed a framework that models the therapeutic planning processes of a human health advisor during a counseling session. The core of the framework was an OWL ontology of health behavior change concepts. Two behavioral interventions were modeled using the framework: the first to promote physical activity (walking) and a second to promote fruit and vegetable consumption.

People learn differently, with some relying predominantly on the textual presentation of information while others learn by doing. Among several learning theories, the VARK presentation model is appropriate for patient education [37]. The acronym VARK stands for different learning modalities: V(isual) is preferred by learners who absorb information through pictorial representation, A(ural) is preferred by learners who absorb information through audio, R(ead/write) is preferred by learners who absorb information through textual representation, and K(inesthetic) is preferred by learners who absorb information by practicing or manipulating things. Customizing educational materials along these learning styles can stimulate and sustain patients’ attention when learning about their conditions or treatment options.

The process of teaching is often guided by Bloom’s taxonomy of Educational Objectives [15], which contains six categories of cognitive skills (remembering, understanding, applying, analyzing, evaluating, and creating). These are often simplified into two levels of complexity: *lower-order learning*, which requires less cognitive processing, and *higher-order learning* [38], which requires a greater degree of cognitive processing. Bloom’s taxonomy [15] has been widely used in the development of ITS to automate the personalization of education materials by navigating between comprehension levels, adapting the materials to be less/more difficult depending on the learner’s skills [39,40,41].

The taxonomy is also used in medical education frameworks for clinicians, when teaching the diagnostic and treatment process [42,43] and as a tool to help nurses transfer medical information to patients and their families during education sessions [44]. However, the taxonomy has not been directly employed in the development of educational materials for patients. In this work, we propose to use Bloom’s taxonomy to address the research gap of personalizing patient education materials to the skills of different learners (patients) by generating educational materials from an ontology that explicitly encodes domain knowledge for a clinical condition, as well as health education content that matches the skills of different learners. We do so by focusing on two clinical use cases, namely Atrial fibrillation (AF) and lower back pain.

AF is the most common arrhythmia in the general population and its prevalence varies with age; approximately 1% in people under 60 years old and approximately 8% in people over 80 years older suffer from AF [45]. Patients with persistent AF receive medication to control heart rhythm and/or rate and are given anticoagulation medication, such as warfarin, to minimize chances of stroke and systemic embolism. Unfortunately, a large proportion of AF patients take anticoagulation medication inconsistently or entirely give up this line of treatment [46]. Thus, knowing about AF-related risks and understanding how anticoagulation medication should be taken is very important for better adherence to the prescribed treatment.

Lower back pain is one of the most common reasons people seek medical advice, and it is a leading cause of disability worldwide [47]. It is often a symptom of a lumbar degenerative disc condition associated with ageing. The most indicative symptom of a degenerated disk is a low-grade, continuous pain that occasionally flares up into more severe, potentially disabling pain. Improving physical condition and exercising is one of the most effective ways for avoiding back pain or preventing its recurrence. Patients experiencing lower back pain should fully understand the physiology of the pain and treatments/interventions that mitigate that pain.

## 3. Materials and Methods

Our proposed framework represents a new eLearning tool to enhance patient knowledge about and adherence to treatment. Using several representative use cases, we conduct a mostly qualitative study supported by a quantitative questionnaire to investigate the acceptability of the tool among the target patient population and medical practitioners. The framework is inspired by ITS and uses an ontology (*eLearning ontology*) to express domain (condition and treatment) and learner (patient) models. It personalizes educational materials to the patient’s characteristics, teaches patients how to manage their treatment within daily life constraints, and provides a means to independently solve issues such as missed medication doses. It creates a personalized *course* composed of the *lessons* that cover all the knowledge associated with a patient’s condition and treatment options, customized to a patient’s skills (i.e., learning capabilities), learning styles, and their progress.

The lessons deliver *basic units of knowledge* (BUKs) that represent an atomic chunk of knowledge about a condition, treatment, and related concepts. To make the learning experience interactive, we consider two types of BUKs. The first is a *Content BUK*, which delivers definitions of clinical concepts using different modalities. The second is a *Q&A (Question and Answer) BUK*, which is delivered in the form of multiple-choice questions, and is used to assess patients’ understanding, thereby enabling them to reason over the content to problem-solve new situations. The complexity of both types of BUKs is modeled according to the patients’ preferred learning styles and Bloom’s taxonomy. We use the simplified two-level taxonomy, which is best suited for assessment with multiple choice questions [38].

### 3.1. eLearning Ontology

**Figure 1:**
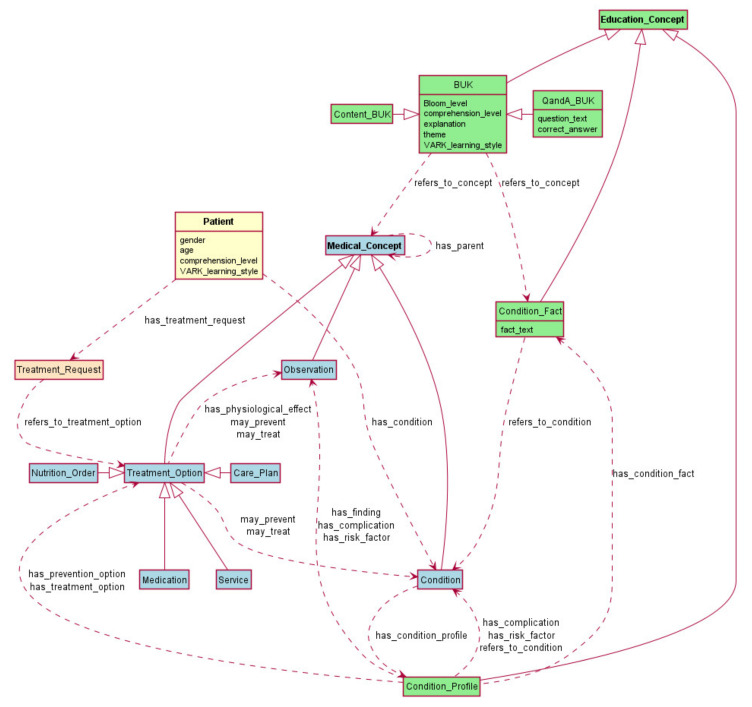
eLearning ontology (selected auxiliary concepts and relations have been removed for clarity).

We use Protégé [48] to specify the OWL [49] eLearning ontology. Figure 1 presents the key classes in this ontology, with the three top-level classes being ***Medical_Concept***, ***Patient***, and ***Education_Concept***.

The ***Medical_Concept*** class stores the preferred name and code taken from a controlled clinical vocabulary (e.g., SNOMED-CT). It has three subclasses: ***Condition***, ***Observation***, and ***Treatment_Option***. The names and meaning of these classes are based on HL7 Fast Healthcare Interoperability Resources (FHIR) [50]. Therefore, the ***Condition*** class “is used to record detailed information about a condition, problem, diagnosis, or other event, situation, issue, or clinical concept that has risen to a level of concern” [50]. The ***Observation*** class stores signs, symptoms, laboratory test results, and imaging results associated with conditions. The ***Treatment_Option*** class specifies the treatment and its physiological effects and points at the conditions that it may treat or prevent via the *may_treat* and *may_prevent* properties. Following FHIR’s classification, ***Treatment_Option***(s) are specialized into ***Medication***, ***Service***, ***Nutrition_Order*** (diet), and ***Care_Plan*** (e.g., a physical activity plan).

The ***Patient*** class has a property ***comprehension_level***, which is related to a patient’s comprehension level of their health care. Because it is very difficult to obtain this comprehension level directly, we use patients’ level of education as a proxy (higher educational attainment usually predicts a higher comprehension level [51,52,53]). For simplicity, we assume that there are three levels: low (associated with patients without a high school diploma), medium (associated with a high school diploma), or high (associated with higher educational attainment). Another property of a patient is their preferred ***VARK_learning style***(s), which are the V, A, R, or K learning styles, and a patient may specify more than one preferred style.

A ***Patient*** also has a medical ***Condition***(s) and respective ***Treatment_Request***(s) (inspired by FHIR) that specify medication dosage information (dose, rate, and timing of the doses) and refer to ***Treatment_Option***(s) from FHIR, such as ***Medication***, ***Nutrition_Order***, ***Service***, or ***Care_Plan***). The data types used in the ontology conform to the standard FHIR data types. The primitive temporal elements of FHIR (i.e., time, date, dateTime, instant) and the general-purpose temporal elements (i.e., period, range, duration) can be mapped to respective OWL Time Ontology classes. In addition, FHIR has complex types for expressing repeating timing information (Repeat resource).

**Figure 2:**
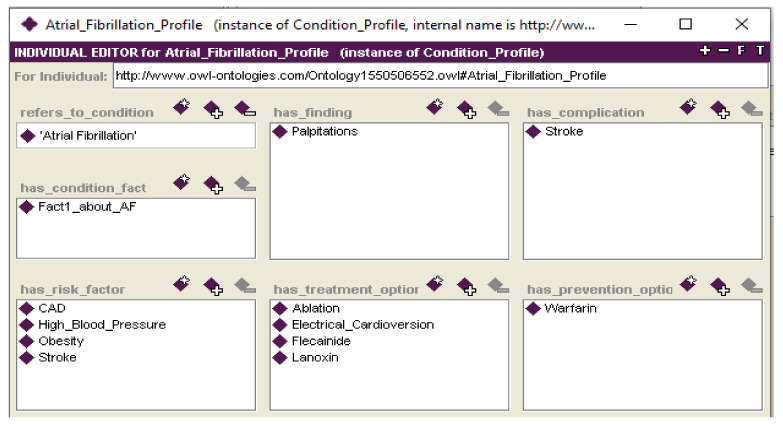
***Condition_Profile*** concept for Atrial Fibrillation.

The ***Education_Concept*** class stores educational materials and has three subclasses: ***Condition_Profile***, ***Condition_Fact***, and ***BUK***. Given the focus on condition, a ***Condition_Profile*** (see Figure 2) details the properties regarding a Condition on which a patient should be educated. The properties of the ***Condition_Profile*** class are organized according to the topics derived from online health sources such as WebMD or the Center for Disease Control. These topics include condition facts, findings (related symptoms, signs, laboratory test results, radiology findings), complications, risk factors (which may be related to conditions or findings), treatment options, and prevention options. To standardize the terms used in the ontology, the allowed values of the ***Condition_Profile*** properties are ***Medical_Concepts***, which provides a controlled vocabulary code, and ***Condition_Facts***, which allows recording various facts about the condition that are not associated with a specific controlled terminology.

**Figure 3:**
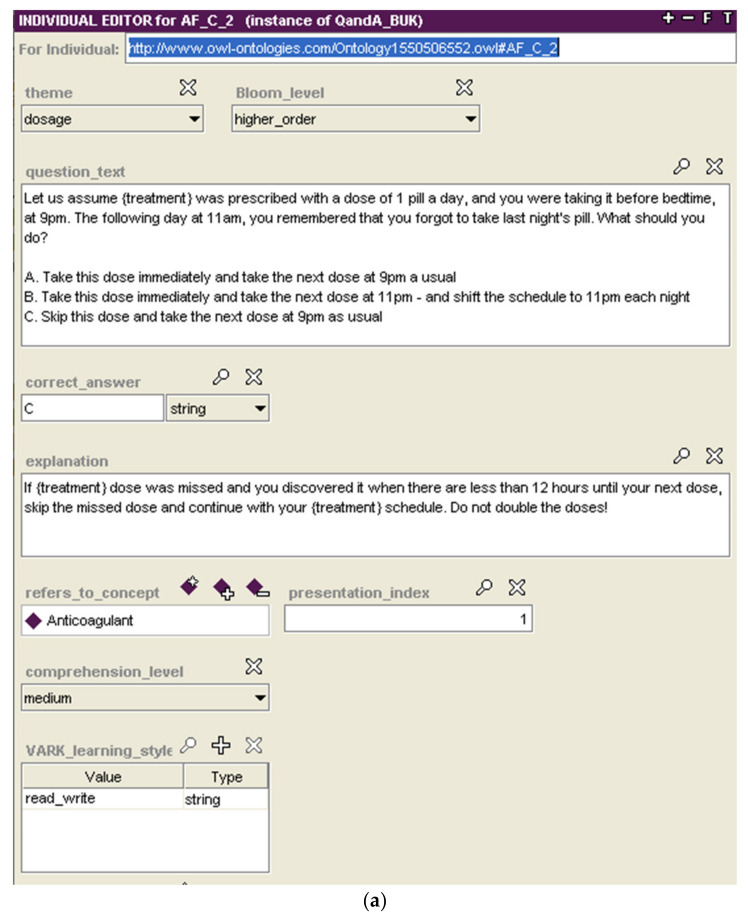

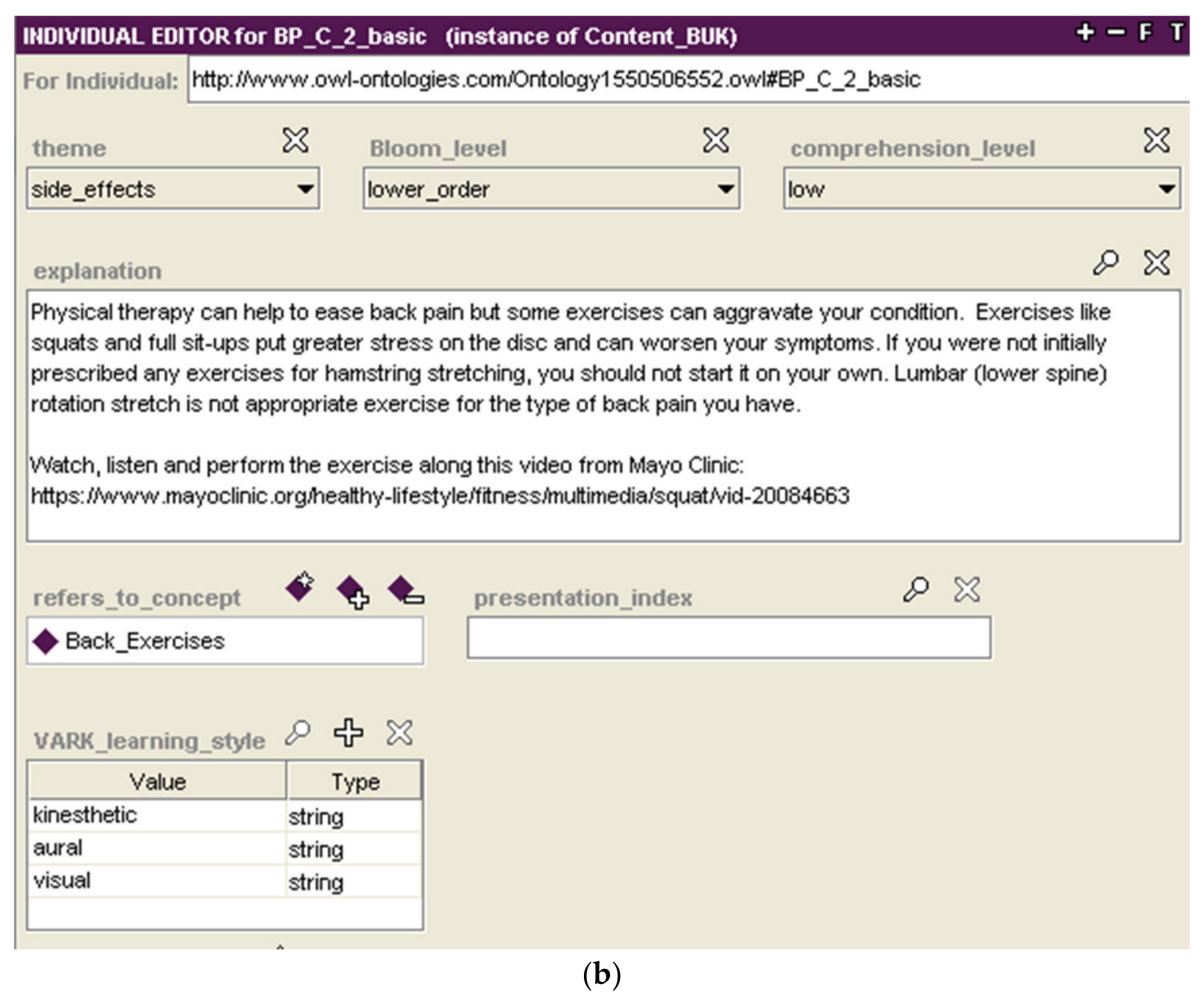
Examples of BUKs represented in the eLearning ontology. (**a**) A Q&A BUK related to the missed anticoagulant dosage annotated for higher-order learning; (**b**) A Content BUK related to the exercises that are not recommended for patients with back problems annotated for low comprehension level.

For each property of a ***Condition_Profile***, the eLearning ontology provides a set of BUKs. Each BUK refers to a single ***Medical_Concept*** (via *refers_to_concept* property). For example, the BUK shown in Figure 3a refers to anticoagulation medication and that of Figure 3b refers to back exercises therapy. Note that the BUK in Figure 3a contains the “{treatment}” variable, which is replaced with the name of a specific anticoagulation medication (e.g., warfarin) when the BUK is presented to a specific patient.

BUKs refer to concepts that are at a different level of generalization. Specifically, ***Medical_Concepts*** constitute hierarchies defined using the *has_parent* relation, and BUKs are associated with concepts at different levels of these hierarchies. In this way, we create personalized lessons for patients that address general principles (e.g., how to cope with missed dosage of any kind of medication), more specific principles (e.g., how to cope with risky events associated with anticoagulants), and very specific principles (e.g., how to cope with diet restrictions associated with warfarin).

Personalization of educational materials require that each BUK captures specialized information that is further customized to a patient’s characteristics. Thus, multiple BUKs associated with the same ***Medical_Concept*** or ***Condition_Fact*** are specialized according to themes. A theme is represented as a BUK class property and in the case of a BUK associated with a ***Treatment_Option*** (a sub-class of ***Medical_Concept***), the themes are dosage, monitoring tests required to monitor the treatment effects and side effects, direct effects, side effects, risky events, and diet restrictions.

Considering that patients have different capabilities to comprehend information presented by a BUK, and their learning process is influenced by their VARK learning style, we ascribe BUKs with three additional properties. The first property is the VARK learning style exhibited by the ***explanation*** property of the BUK that captures the main educational content. Of note, the ***question*** property in the Q&A BUK is always associated with the R and A learning styles from VARK. The second property is the ***comprehension level*** required from a patient training with the BUK (low, medium, or high). The third property pertains to educational goals and is the simplified ***two-level Bloom taxonomy*** associated with the level of complexity of information provided by a BUK.

Because OWL does not support complex sequencing of elements [54] such as imposing order on properties and their values, which are crucial for creating a sequence of lessons forming a course, we rely on a control and execution mechanism with precompiled plans based on the principles of a Procedural Reasoning System (PRS) [16] (see Section 3.2). Specifically, precompiled plans define the scope and sequencing of lessons within a course, the sequencing of BUKs within a lesson, and the control interaction with the patient (learner) when presenting lessons by displaying BUKs, capturing responses, and evaluating them. Precompiled plans are processed by the planning and execution module of the framework (discussed below), and they are combined with the content of the knowledge base derived from the eLearning ontology to generate a personalized course. Within each course, lessons contain BUKs that match the patient’s comprehension level and the level of Bloom’s taxonomy of educational objectives.

### 3.2. Planning and Execution Framework

Our framework draws from PRS [16], which was proposed as the control architecture for intelligent software agents and uses a library of precompiled plans. A precompiled plan specifies the goal it achieves, preconditions that need to be satisfied so the plan can be invoked, and the body of the plan that contains specific procedural steps. A goal may be parametrized to better control the execution of a precompiled plan. For example, the goal of developing and delivering a course has three parameters: a patient, a condition, and a treatment. Preconditions are optional, and if they are not explicitly defined, they are automatically satisfied, and a precompiled plan is always invoked. Finally, a precompiled plan may introduce additional goals if they need to be achieved prior to the current goal—this results in pausing the execution of the current plan and invoking other precompiled plans associated with these additional goals.

We use precompiled plans to establish the sequence of lessons constituting a course and the sequence of BUKs within a lesson, and to control the presentation of specific BUKs. A precompiled plan for sequencing lessons takes advantage of the fixed general structure of a course captured by the ***Condition_Profile*** concept—its properties are considered always in the same sequence, resulting in a simpler procedural body. A precompiled plan for sequencing BUKs within a lesson has a more complex body as it needs to identify and retrieve BUKs appropriate for a particular patient (e.g., corresponding to their comprehension level and a preferred learning style). Moreover, the possibility of invoking other plans from the plan body allows splitting the process of course development and delivery into smaller segments that are easier to maintain and update.

**Figure 4:**
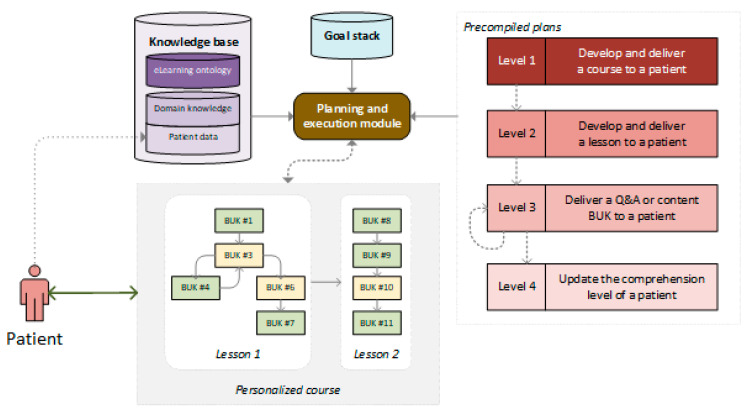
Architecture of the planning and execution framework for developing and delivering personalized courses.

The architecture of our framework for developing and delivering personalized courses is presented in Figure 4. The principal architectural components—planning and execution module, goal stack, and knowledge base—come from PRS and have been adapted to our specific problem.

The **knowledge base** stores the eLearning ontology and instances of concepts from this ontology that capture the domain knowledge and the patient data. The latter includes basic demographics, prescribed treatments, and the performance log that tracks the patient’s learning progress and responses to questions from Q&A BUKs. The domain knowledge is stable and changes infrequently, while the patient data may change during the learning process. The **goal stack** stores goals with the currently pursued goal on top. For a patient diagnosed with a specific medical condition and prescribed treatment for this condition, the initial goal is to develop and deliver a personalized course to improve the patient’s understanding of their condition and treatment. The **planning and execution module** selects a precompiled plan by matching its goal to the current goal from the goal stack and checking if its preconditions are satisfied in the knowledge base. When the plan is selected, its body is executed and possible procedural steps include retrieving content from the knowledge base, displaying information to the patient, capturing and evaluating responses from the patient, and adding new goals to the goal stack (i.e., a plan responsible for developing and delivering a lesson adds the goals of presenting specific BUKs).

The precompiled plans that we used are summarized in Table 1. They are grouped into four levels depending on the goal they satisfy (and, thus, the aspect of the course development and delivery process they handle). There are single plans at Levels 1, 2 and 4, and two plans at Level 3 that deal with delivering both types of BUKs (content and Q&A). Questions and explanations from BUKs are presented following the preferred VARK learning style of the patient (i.e., styles of the patient and BUK are matched). We set an initial style for each patient and they can change the presentation mode of delivered BUKs when others are available. If a given BUK is not available in the preferred style, then the K → V → R → A sequence of styles is considered, and the first supported style is used. As noted earlier, Q&A BUKs are available only in A or R modes, while Content BUKs can be presented in any of the VARK modes.

The sequencing of lessons in the Level 1 plan and themes in the Level 2 plan follows conventions used in WebMD. To avoid repeating the same Q&A BUK in the Level 3 plan when a patient fails to provide a correct answer, it is possible to predefine in the knowledge base a sequence of Q&A and Content BUKs for a given concept and theme (this simple sequence can be specified in OWL by assigning indexes to BUKs). If such a sequence is specified, subsequent BUKs from the sequence are delivered, and the entire sequence is repeated if no correct answer is given by a patient.

### 3.3. Proof-of-Concept Implementation

To create the personalized course for a patient, we interface with the Electronic Health Record (EHR) used at the point of care. We rely on well-established standards such as HL7 FHIR to receive notifications about patients being diagnosed with new conditions and treated for these conditions. After receiving such a notification, we create instances of the *Patient* and *Treatment_Request* classes in the knowledge base and link the *Patient* instance to the appropriate *Condition* instance. If the EHR does not provide any data about the patient’s comprehension level, we prompt the attending physician to provide this information based on their judgement. This is the only place where physician involvement is necessary. However, given recent initiatives to extend the scope of EHR to social and behavioral domains [55], this involvement may soon be reduced even further.

**Figure 5:**
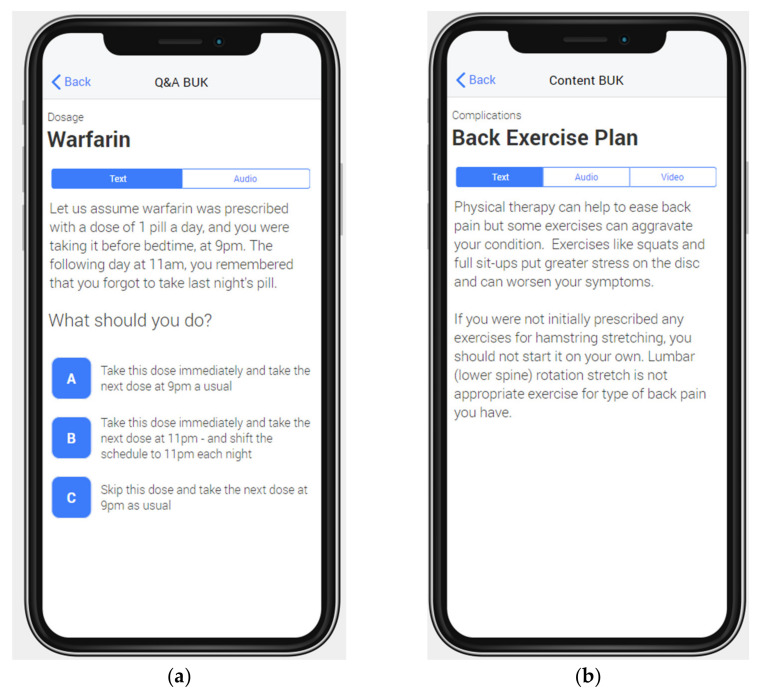
(**a**) A Q&A BUK related to missed anticoagulant dosage for higher-order learning customized for warfarin; (**b**) A Content BUK regarding back exercises for patients with a low basic level of comprehension and the Read/write VARK presentation style.

Once the knowledge base is updated, the patient uses a dedicated front-end (a mobile application) to access the course and to take lessons. While the scope and outline of the course is decided in advance (see previous section), BUKs constituting specific lessons are selected in real time to respond to patients’ learning progress. We developed a proof-of-concept patient-facing mobile application to instantiate our framework for the two use cases described in Section 3.5. Figure 5 shows both a Q&A and Content BUK for the Atrial fibrillation and lower back pain use cases, respectively. These BUKs are generated from their representation in the eLearning ontology, specifically those shown previously in Figure 3, and are available in different modalities.

User-centered design asks for active involvement of potential end-users in a process of designing interactions and presenting content (BUKs). As such, the proof-of-concept mobile application was created in consultation with the stakeholders in our research team—three clinical specialists (two hematologists and one spine surgeon) and six patient representatives (three being treated with anticoagulants and three being treated for lower back pain).

### 3.4. Course Generation

When generating personalized courses for the use cases described in Section 3.5, we highlight several capabilities of our framework. We demonstrate how a sequence of Q&A and Content BUKs related to the same concept, theme, and order of learning is delivered when the patient fails to provide correct answers. We avoid repeating the same Q&A BUK multiple times, which would result in a patient guessing the right answer by elimination. We present general knowledge (e.g., associated with general concepts of dosages of any anticoagulation medication) to give the patient a broader understanding of a treatment. In subsequent interactions, a patient is presented specific textual information that focuses on dosing. VARK learning styles are implemented by allowing the patient to access information about the interactions between treatments in a multi-modal manner (textual, visual, visual, and kinesthetic), reflecting the patient’s learning styles. A generated course also progresses across Bloom levels by first delivering lessons focused on remembering (lower-order learning), and only after several correct answers, it proceeds to delivering lessons aimed at understanding and applying the acquired knowledge in practice (higher-order learning).

### 3.5. Evaluation Methods

We carry out a mixed methods study of the acceptability of the tool among the target patient population and medical practitioners, combining a qualitative approach supported by a quantitative questionnaire. The user-centered design relies on the end-users providing feedback on different aspects of the information system and a design team rapidly implementing this feedback (rapid prototyping) for subsequent assessment by the end users. To facilitate this process, we developed two scenarios. The first concerns anticoagulation treatment associated with the management of an AF condition. It describes the development of a personalized course for a fictional patient “Mario,” who was recently diagnosed with AF and prescribed warfarin as anticoagulation medication. Mario’s preferred VARK learning styles include V and R.

The second scenario pertains to back pain management to treat a lumbar degenerative disc condition. In it, we consider a fictional patient “Anne,” who is suffering from chronic, non-specific back-dominant pain and was recently diagnosed with a lumbar degenerative disc condition. Anne is an active learner who prefers to absorb information either in a V or K style. According to the guidelines [56], Anne was prescribed daily maintenance dosage of NSAID (ibuprofen) and was given a set of twice-daily back strengthening exercises. Courses for both use cases were created using the planning and execution module described in Section 3.2. The module is implemented in Python and uses the Owlready2 library [57] to access the eLearning ontology stored in the OWL file.

To study the acceptability of the proof-of-concept implementation for each scenario, we asked our collaborators (patient representatives and clinical specialists) to provide answers to a questionnaire and to supplement those answers with qualitative explanations and insights. We created a questionnaire derived from the Technology Acceptance Model questionnaire [58]. The same questionnaire was given to all collaborators; however, patient representatives received slightly revised descriptions of the scenarios. The questionnaire contained eight questions. The first five questions use quantitative scales with response options ranging from 1 (strongly disagree) to 5 (strongly agree). The sixth question has a quantitative scale of 0, 1, and 2, representing No, Unsure, and Yes, respectively. The last two questions have open-ended text inputs that elicit the expected mode of usage of and any suggested changes to the proof-of-concept implementation. After being presented with a scenario, reading an abridged description of the courses developed using the framework, and seeing mockups of the interface, each of the collaborators was asked to answer the quantitative questions and provide a qualitative explanation of their answers. We also solicited any additional feedback and suggestions regarding content and interactions to be implemented in the proof-of-concept mobile application.

## 4. Results

The application of our framework results in a personalized course for each patient scenario. Table 2 outlines Mario’s interaction in the AF use case (assuming a high comprehension level), while Table 3 outlines Anne’s interaction in the lower back pain use case (assuming a low comprehension level).

**Figure 6:**
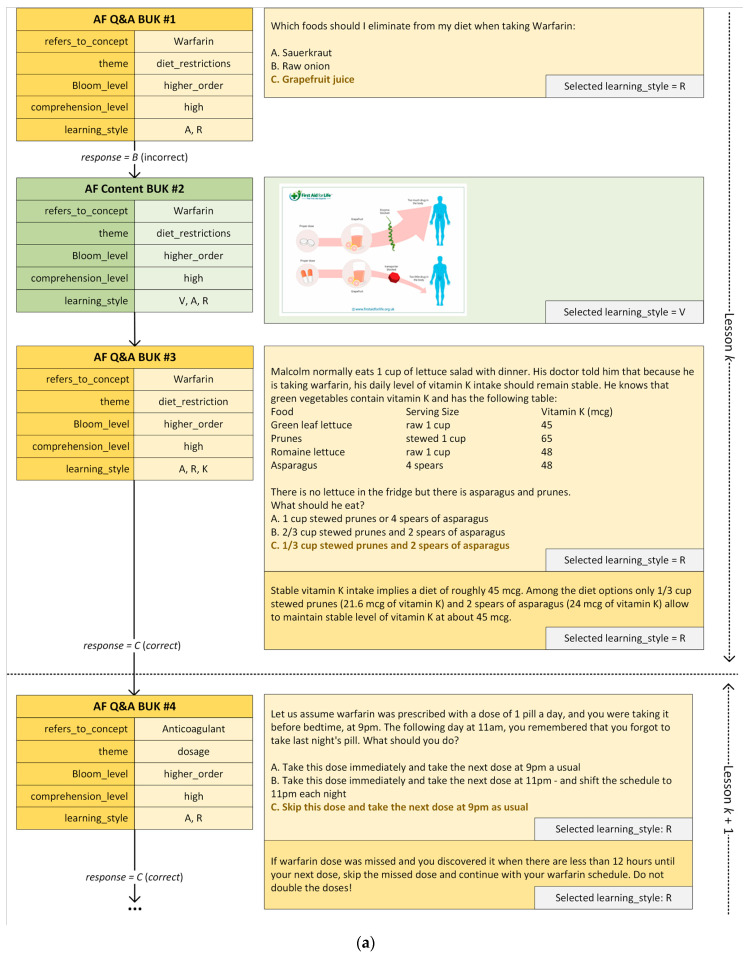

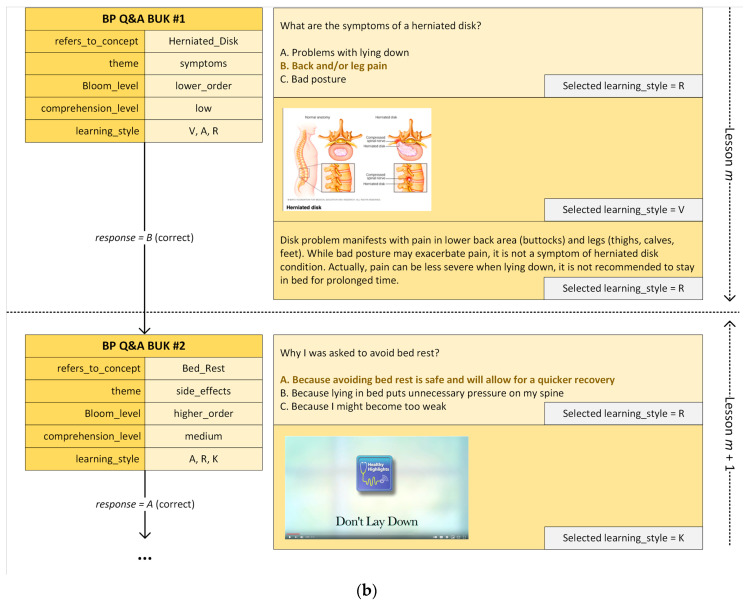
Selected lesson fragments for the two use cases: (**a**) anticoagulation treatment associated with AF and (**b**) lower back pain management required for treating lumbar degenerative disc condition.

Figure 6 illustrates the sequence of interactions and the development of the personalized course including text associated with each BUK generated by our framework. Q&A and Content BUKs are indicated with orange and green boxes, respectively. Arcs indicate transitions between BUKs. For transitions starting at Q&A BUKs we indicate whether a provided answer was correct or not.

Table 4 provides a summary of the quantitative responses, while Table A1, Table A2 and Table A3 in Appendix A provide the detailed qualitative responses from our collaborators. We analyzed the responses together with the feedback provided, and if necessary, revised content and presentation of the BUKs.

Responses to our questionnaires show that both end-user groups were positive about our proof-of-concept and liked how it structures the learning process. Overall, clinical specialist end-users saw more advantages from having personalized educational materials as compared to the patient representatives. This was especially true for the questions around personalization of the educational materials. While clinical specialists perceived “personalization” to mean creating unique materials for a specific group of patients, some of the patient representatives interpreted “personalization” to mean receiving materials customized just to an individual patient.

These differences in interpretation likely result from the fact that physicians and patients differently perceive what it means for an educational material to be “personalized”—something that we did not envisage while developing the questionnaire. A physician sees many patients with the same condition, and they want to “personalize” education to a homogenous sub-cohort of these patients. On the other hand, a patient is the one with a specific condition and does not see the larger relatively homogenous group of their “peers” with the same condition and similar health status. Thus, for a patient the course is “personalized to them” while for a physician it is “personalized to a homogenous group.” In future iterations, patients will be educated on what is meant by personalization.

Both end-user groups considered our proof-of-concept easy to use. This is best summarized by one of the clinical specialist collaborators, who commented that “lessons are intuitive and easy to follow” and a patient representative collaborator who said that “the interactive structure is more interesting and relevant than googling and reading long articles. Multiple choice Q&As are more fun. Info presented in this interactive way and in shorter sections is also easier for most people to retain.”

Comments about the usefulness of the proof-of-concept were equally interesting. In one patient representative’s opinion “offering personalized materials is a great way to better education [sic] patients about their condition and how to manage it. There is a lot of information out on the internet that people can read and interpret in different ways,” while another noted “I know I would like such a tool […] It has always been very frustrating for me that the only way to obtain personalized information about my back issues is to go to a physio/chiropractor/doctor. They are obviously the first and very important line of support, but one doesn’t go to these therapists indefinitely, nor is one able to ask them questions between visits. Even at a visit one doesn’t necessarily ask the right questions or properly take in all the information presented.”

In turn, clinical specialists saw our proof-of-concept as facilitating/supporting a patient encounter: “Personalized information will allow patients to better understand their condition, have more productive discussions with their physician and potentially decrease the need for physician visits” and “It is hard for clinicians to go through all the QAs in one encounter with the patient. Furthermore, different patients may have different questions or concerns which need to be addressed. Finally, patients often will only remember of questions once back home. Having access to personalized materials that they can have access to is important.”

In terms of how our proof-of-concept would be used in practice, two viewpoints emerged and are best illustrated by these quotes. First, from a clinical specialist’s viewpoint: “It will be reassuring for patients to have access to a personalized teaching framework. [They] would probably go to it before contacting their health care provider,” and second, from a patient’s viewpoint: “For me this would be about learning about my back, the origins of the pain in my particular case; then understanding why health care professionals are recommending certain exercises/therapies/drugs (how will those help), and what my part is in helping to ensure these are effective. […] The teaching framework could also provide useful knowledge and info about how to best describe the pain and what questions to ask when I do see a health care professional.” These two seemingly disconnected viewpoints align with the single goal of better understanding a condition and the treatment plan. During this participatory process, the end-user collaborators also suggested changes to the wording (e.g., identifying words that were too complex) that we incorporated into the proof-of-concept implementation discussed in the paper.

## 5. Discussion

In previous work [9], we proposed an ideating approach grounded in behavioral theories that motivates and engages patients, and this proposed framework builds on our earlier findings. That earlier work focused on different activities that can make patients better engaged and compliant to their therapy by increasing patients’ motivation and competence. Those activities included: goal setting, barrier detection and mitigation, action planning, reporting of symptoms and progress in becoming engaged, and consulting with a daily and weekly summary that tracks physiological outcomes and compliance to recommendations and compares compliance to normative and peer compliance levels. However, our previous work presented educational materials in a non-personalized and non-interactive way. The current work constructs courses personalized across four patient axes—condition and treatment, comprehension level, learning style based on the VARK presentation model, and the level of understanding of specific course content according to Bloom’s taxonomy. Furthermore, the presentation of Q&A BUKs creates an engaging experience as noted by the patients and clinical experts.

The eLearning ontology represents the breadth of medical and educational concepts and their relationship. It draws its generality by building on standards such as HL7 FHIR, SNOMED-CT codes, and condition profiles used in online health sources such as WebMD, the Center for Disease Control, and others. It is comprehensive enough to be used for different conditions and operationalizes an ontology-driven design for personalization of educational materials for a patient. The uses cases that we selected were much different from each other in terms of therapy options. While the AF example focused on drug therapy, the lower back pain management focused on physical exercise therapy. Yet, the same construct of *Treatment_Request* is used to denote the personalized recommendation for the patient that includes the therapy type (e.g., warfarin, yoga) and the dosage (e.g., 1 tablet/day, 1 metabolic unit/3 times a week).

The use cases also demonstrate the opportunity for reusing some content for different patients with different conditions. For example, Figure 2 shows the *Condition_Profile* of the AF condition with stroke as one of its complications. Hence, BUKs created for the stroke condition can be reused when developing a course for patients suffering from AF. The same applies for BUKs relating to findings (i.e., signs and symptoms). Generic BUKs that teach about dosage and what to do when a dose is missed can be reused for other drug therapy options. For example, BUK #4 (Figure 6a) is applicable as educational material about anticoagulants and with a minor modification in the listed medication names could be reused when developing a course that involves education about antiplatelet (such as dopidogrel or ASA) or cholesterol-lowering (such as statins) medication. The same applies to general recommendations about diet and exercise. As our use cases show, the eLearning ontology can be instantiated without making assumptions about the specific patient condition or the type of educational material to present.

Finally, these use cases show the power of tailoring presentation styles to patients’ preferred learning style. Incorporating the VARK presentation model into the eLearning ontology supports customized presentation modalities for both content and Q&A BUKs that can change over the course of a lesson. Different content lends itself to different presentation modalities. Presenting content with a consistent modality and giving patients the ability to change the modality when possible is both engaging for the patients and caters to the breadth of patients’ learning styles.

Our framework combines an ontological representation of knowledge with reasoning, in the form of planning and execution. It differs from existing approaches as it relies on precompiled plans and an ontology while others typically use rules. Our approach offers more flexibility and allows for more complex course strategies for course development and delivery than only rule- or ontology-based approaches. For example, it is difficult to handle more complex sequencing in OWL. While there have been attempts to achieve it [54], they require expanding the ontology with additional concepts and properties, which introduces unnecessary complexity to knowledge representation. Our proposed approach supports sequencing natively with no need for the introduction of auxiliary concepts and mappings and we rely on this ability to establish the sequence of lessons within a course, and the sequence of BUKs within a lesson. Moreover, introducing multiple precompiled plans responsible for specific aspects of course development and delivery facilitates defining and maintaining these plans. As already discussed, it is easy to revise selected plans to change how BUKs are sequenced within a lesson or how they are delivered to the patient.

We are very encouraged by the feedback received from collaborating end-users (clinicians and patient representatives) who were involved in the proof-of-concept implementation, as it shows that our proposed solution is a step in the right direction. Two future applications for our framework emerged—one from the physician’s and one from the patient’s viewpoint. From a physician’s perspective, the framework can be used to create a course that will prepare patients for their meeting with specialists by educating them about their condition and helping them formulate questions to ask during the encounter. From the patient’s perspective, the framework can be used to create a course tailored towards learning more about their prescribed treatment and provide them with additional information describing the progression of this treatment.

## 6. Conclusions

In this paper, we present a novel methodological framework for developing a personalized course to teach patients about their medical condition and associated treatment. We uniquely combine an eLearning ontology with precompiled plans to allow for personalization across levels of comprehension, learning styles based on the VARK presentation model, and Bloom’s taxonomy. We demonstrate the framework’s generality and versatility using two use cases and assess its validity and perceived usefulness with collaborators, including three clinical specialists and six patient representatives. Our research contributes to the development of methods and tools that help patients adhere to their therapy.

Our proposed framework relies on several assumptions that may be considered as limiting factors. These assumptions are made internally before using the framework to develop a course and are unknown to the patient assigned a course:

- The structure of a course is fixed and progression through a course is linear;
- A patient’s comprehension level is known in advance;
- Only a Content BUK and the explanation part of a Q&A BUK are associated with multimodal presentation;
- A patient’s learning style is set to a default and not determined a priori, and as such, a patient is free to change the mode of presentation for each BUK separately;
- BUKs are fixed and they need to be developed in advance for different comprehension levels.

To address the above limitations, our future work will focus on several key features. First, we will allow patients to select the starting point within a course and provide them with the ability to start learning about a specific concept within a lesson. Second, we plan to develop rewind and fast-forward functionality to support an additional layer of learning and customizing lessons to patients’ learning styles. Third, we will update the model of a BUK to be a template so their textual content can be customized to the patient as the course is being assembled. Fourth, we will learn a patient’s preferred VARK presentation mode through their interactions with BUK presentation modalities and consider using one of the measures provided by the Health Literacy Toolshed (https://healthliteracy.bu.edu/, accessed on 7 July 2021) to establish a patient’s comprehension level. Finally, we will design and implement a pilot study for multiple disease conditions and involving actual patients. We will test patients’ ability to retain knowledge about their treatment and their adherence to this treatment using the mobile application at home for a longer period. We will then evaluate the uptake of our application at different time points during the study.

## Figures and Tables

**Table 1:** Precompiled plans used in the framework.

**Level 1 Plan**	
Goal:	Develop and deliver a personalized course to a patient
Parameters:	Patient *P*, diagnosed condition *C*, prescribed treatment *T*
Preconditions:	None
Body:	Iterate over properties of an instance of the ***Condition_Profile*** concept (see Figure 2) associated with condition *C* to establish the sequencing of lessons. Select medical concepts or condition facts included in a current property to establish the scope of a lesson defined as a set of medical concepts or condition facts. When considering treatment options, limit them to treatment *T*. Add a new goal “develop and deliver a lesson with established scope to a patient” with patient *P* and add the current lesson scope as parameters to the goal stack.
**Level 2 Plan**	
Goal:	Develop and deliver a lesson with an established scope to a patient
Parameters:	Patient *P*, lesson scope *S*
Preconditions:	None
Body:	Iterate over condition facts or medical concepts included in the scope *S* (called scope items) to establish the sequencing of BUKs in a lesson. If the current scope item is a medical concept that represents the treatment or prevention option (i.e., is an instance of the ***Treatment_Option*** concept), then iterate over themes and Bloom levels. Otherwise, determine the Bloom levels only (technically, the wildcard “any theme” is used). If a current scope item is a child medical concept (it is associated with more general concepts using the *has_parent* property), then explore the hierarchy of concepts in the top-down direction. For the current scope item (concept or fact), theme, and Bloom level, retrieve a BUK *B* that matches the comprehension level of the patient *P*. If such a BUK does not exist, then retrieve a BUK for a lower level. Add a new goal “deliver a BUK to a patient” with patient *P* and the retrieved BUK as parameters to the goal stack.
**Level 3 Plans**	
Goal:	Deliver a BUK to a patient
Parameters:	Patient *P*, BUK *B*
Preconditions	*B* is a Content BUK
Body	Customize the explanation of the BUK *B* to the current treatment if necessary. Present the explanation according to the preferred VARK learning style for the patient *P* (allow *P* to change the style of the explanation).
Preconditions	*B* is a Q&A BUK
Body	Customize a question from the BUK *B* to the current treatment if necessary. Present the question according to the preferred VARK learning style for the patient *P* (allow *P* to change the style for the question). Capture a patient’s response. Update the performance log *L* with the response correctness. Add a new goal “update the comprehension level of a patient” with patient *P* and log *L* as parameters to the goal stack. If the response is correct, then present an explanation from the BUK *B* using the preferred style of *P* (allow *P* to change the style for the explanation). Otherwise, retrieve a Content BUK associated with the same concept and theme as *B* and matching the comprehension level of the patient *P*. Once the BUK has been retrieved, add a new goal “deliver a BUK to a patient” to the goal stack. If the response is incorrect and there is a sequence of Q&A BUKs associated with the same concept and theme as *B*, then retrieve the next Q&A BUK from the sequence (if *B* is the last BUK, then retrieve the first one). Add a new goal “deliver a BUK to a patient” with patient *P* and the retrieved BUK as parameters to the goal stack. If the response is incorrect and there is no sequence of related Q&A BUKs, then restart the plan by presenting the BUK *B* again.
**Level 4 Plans**	
Goal:	Update the comprehension level of a patient
Parameters:	Patient *P*, performance log *L*
Preconditions	*L* contains at least *n* entries capturing responses to questions from Q&A BUKs
Body	Calculate the trend and average accuracy from the last *n* entries in *L*. If the accuracy is satisfactory and the trend is increasing, then increase the comprehension level. Otherwise, if the accuracy is not satisfactory and the trend is non-increasing, then decrease the comprehension level. Update the comprehension level for the patient *P*.

**Table 2:** Interactions between Mario and the framework in the AF use case (high comprehension level, preferred learning style: V and R). For the identifiers of Q&A and Content BUKs, refer to Figure 6a.

#	Interaction Description
1	Mario is presented a Q&A BUK (AF Q&A BUK #1) for diet restrictions while taking warfarin and associated with Bloom’s higher-order learning.
2	Mario provides an incorrect answer. However, due to his good performance track record (majority of his recent response were correct), Mario’s comprehension level is not changed.
3	Mario is presented a Content BUK (AF Content BUK #2) with additional explanation.
4	Because of the incorrect answer, Mario is presented with a different Q&A BUK (AF Q&A BUK #3) that is associated with the same concept, theme, and order of learning.
5	Mario provides the correct answer and is presented an additional short explanation to reinforce his learning.
6	Mario is presented a Q&A BUK (AF Q&A BUK #4) on dosing anticoagulant medication. Note that while this BUK is of a general nature and applicable to multiple anticoagulant medications, it has been customized for warfarin.
7	Mario provides the correct answer and is presented an additional short explanation to reinforce his learning.

**Table 3:** Interactions between Anne and the framework in the lower back pain use case (low comprehension level, preferred learning style: K and V). For the identifiers of Q&A and Content BUKs refer to Figure 6b.

#	Interaction Description
1	Anne is presented with a Q&A BUK (BP Q&A BUK #1) pertaining to symptoms of lumbar degenerative disk condition and associated with Bloom’s lower order learning.
2	Anne provides a correct answer and is presented with an additional short explanation in her preferred style. She also requests the explanation in the R style to further reinforce her learning. Moreover, due to her good recent performance, Anne’s comprehension level is promoted to medium.
3	Considering that Anne demonstrated a grasp of knowledge associated with a lower level of learning and that her comprehension level improved, a Q&A BUK associated with a higher-order learning and medium level (BP Q&A BUK #2) is presented to her.
4	Anne provides a correct answer and is presented with an additional short explanation to reinforce her learning.

**Table 4:** Summary of the responses of collaborating end-users to the TAM-inspired questionnaire.

**Perceived Ease of Use**							
		1	2	3	4	5	Mean
1. The purpose of the lessons will be clear to patients?	Physicians				1	2	4.67
	Patient representatives			1	4	1	4.00
2. The structure (Q&A followed by Content BUK) of the lessons will be clear to patients?	Physicians					3	5.00
	Patient representatives			1	1	4	4.50
3. The transitions between questions and explanations are intuitive and will be understandable to patients?	Physicians					3	5.00
	Patient representatives			1	3	2	4.17
**Perceived Usefulness**							
		1	2	3	4	5	Mean
4. The content of the lessons is appropriately tailored to different level of health literacy?	Physicians			1	1	1	4.00
	Patient representatives			2	2	2	4.00
**Perceived Intention to Use**							
		1	2	3	4	5	Mean
5. Having access to personalized materials via lesson can help patients to better manage their condition?	Physicians			1		2	4.33
	Patient representatives			1	2	3	4.33
**Recommendation**							
		No	Unsure			Yes	Not answered
6. Would you recommend such personalized teaching framework to every patient?	Physicians		1			2	0
	Patient representatives		1			3	2

Note: The cells show the number of end-users who chose each answer option. The Likert scale for the first five questions was: 1 = strongly disagree; 2 = disagree; 3 = neutral; 4 = agree; 5 = strongly agree.

## Appendix A

**Table A1:** User-centered design: feedback received from clinical specialist end-users’ group.

Question	Clinical Specialist #1	Clinical Specialist #2	Clinical Specialist #3
**Perceived Ease of Use**			
1. Will the purpose of the lessons be clear to patients? (Strongly Agree, Agree, Neutral, Disagree, Strongly Disagree)	Strongly Agree (5). Lessons are intuitive and easy to follow.	Strongly Agree (5). It is a common question for patients. There is a lot of conflicting info on the web, erroneous beliefs, conflicting messages from pharmacists, physicians, relatives, etc.	Agree (4). The lesson on anticoagulants is helping patients be more aware of dietary interactions and troubleshooting medication issues while the back pain lesson is simple and straightforward.
2. Will the structure (Q&A followed by Content BUK) of the lessons be clear to patients? (Strongly Agree, Agree, Neutral, Disagree, Strongly Disagree)	Strongly Agree (5). The structure is clear and easy to follow.	Strongly Agree (5). The fact that the explanation comes just after they answered the question will make it easier to integrate.	Strongly Agree (5). It is very clear that the scenario is followed by an appropriate unit of knowledge.
3. Will the transitions between questions and explanations be intuitive and understandable to patients? (Strongly Agree, Agree, Neutral, Disagree, Strongly Disagree)	Strongly Agree (5). Excellent flow.	Strongly Agree (5).	Strongly Agree (5) although explanation for 1st anticoagulation lesson seems overly detailed. Transition appropriate though.
**Perceived Usefulness**			
4. Is the content of the lessons appropriately tailored to different level of health literacy? (Strongly Agree, Agree, Neutral, Disagree, Strongly Disagree)	Strongly Agree (5). The difference in health literacy is obvious and tailored appropriately to patients’ needs.	Unsure (3). For example, the calculation of actual vitamin K contents might not be appropriate for every patient. Some would likely need more general guidance. Or perhaps you could think of ordering the questions with increasing difficulty, so that patients don’t abandon too early if the questions seem too complex to them.	Agree (4). However, content of 1st anticoagulation lesson seems overly detailed. I would disagree with content in first back pain lesson. Spine practitioners are often trying to re-educate patients that degenerative discs are a normal ageing process and not necessarily a cause of pain. As such, I think this BUK is reinforcing erroneous information. I would change the question to what are the symptoms of a herniated disc…. answer would be back and/or leg pain (sciatica).
**Perceived Intention to Use**			
5. Can having access to personalized materials via lessons help patients to better manage their condition? (Strongly Agree, Agree, Neutral, Disagree, Strongly Disagree)	Strongly Agree (5). It is hard for clinicians to go through all the QAs in one encounter with the patient. Furthermore, different patients may have different questions or concerns which need to be addressed. Finally, patients often will only remember of [sic] questions once back home. Having access to personalized materials that they can have access to is important.	Neutral (3). Not clear how [the material is] ‘personalized.’	Strongly Agree (5). Personalized information will allow patients to better understand their condition, have more productive discussions with their physician and potentially decrease the need for physician visits.
6. Would you recommend such a personalized teaching framework to every patient?	Absolutely. All clinicians are looking for accurate/adequate patient-oriented material to provide. Any resources would be welcomed (but usually need to be “endorsed” by clinicians or clinical societies). Resources should probably also [be] tailored by countries (or culture)?	Yes.	I do think that if teaching materials were tailored that all patients could benefit in some capacity.
7. How do you see patients use such a personalized teaching framework?	Yes. Patient will use it after the initial diagnosis but may also go back to it intermittently afterwards if questions are arising. It will be reassuring for patients to have access to a personalized teaching framework. Would probably go to it before contacting their health care provider.	At initiation of therapy to get general guidance, then perhaps whenever they face a specific challenge.	They could use personalized teaching through their health record (i.e., MYchart) prior to their healthcare visits so they can ask about and discuss their condition with their physician or after the visit to reinforce important points that they learned.
**Other**			
8. Are there any changes or additions to our personalized teaching framework that you would recommend?	No. Looks great.	Proposing sub-topics, so that patients could pick up training that relates to a specific question/training need (e.g., diet vs. missed dose vs. etc.).	As described above.

**Table A2:** User-centered design: feedback received from patient representative end-users’ group for the anticoagulation scenario and a high comprehension level.

Question	Patient 1	Patient 2	Patient 3
**Perceived Ease of Use**			
1. Will the purpose of the lessons be clear to patients?	Strongly Agree (5). The lessons provided were very clear to me and what the extent [sic] was.	Agree (4). Be explicit as to the purpose when supplying the Lesson.	Agree (4).
2. Will the structure (Q&A followed by Content BUK) of the lessons be clear to patients?	Strongly Agree (5). The structure makes sense to me and is logically [sic]. It provides enough information to reinforce the topic.	Agree (4). The manner of delivery stimulates active reading and self-assessment, a desire to learn.	Neutral (3).
3. Will the transitions between questions and explanations be intuitive and understandable to patients?	Strongly Agree (5). Was easy to follow and made logically [sic] sense to me.	Agree (4). The approach reinforces/deepens understanding, and teaches to improved thinking.	Neutral (3).
**Perceived Usefulness**			
4. Is the content of the lessons appropriately tailored to different level of health literacy?	Strongly Agree (5). I agree that the lessons about [sic] are probably for a patient with an expert level of literacy about their condition/disease.	Agree (4). The context is good for an expert literacy level reader. They can absorb and reason through the input.	Neutral (3). I would say this is okay for expert level. Can’t comment on basic level.
5. Can having access to personalized materials via lessons help patients to better manage their condition?	Strongly Agree (5). Yes, I think that offering personalized materials is a great way to better education patients about their condition and how to manage it. There is a lot of information out on the internet that people can read and interpret in different ways. If I got this from my doctor, I would trust that this the information that I should be following to manage my condition	Agree (4). Socratic teaching in small bites when tightly focused on a specific issue captures attention. Insight sinks in [sic] with likely change in behavior resulting.	Neutral (3). Unclear how it is personalized. See final comments.
6. Would you recommend such a personalized teaching framework to every patient?	Yes, I think that this should be offered to patients at the basic and expert level. I think that at the basic level, it would help educate on [sic] why they are taking blood thinners, guidelines to follow etc. For the expert, it’s a good resource for those who want to manage their condition at a high level.	I suggest that you run interviews with two or 3 [sic] patients to test receptivity. Upper moderate to high levels of literacy are necessary for this particular lesson content to work.	Perhaps.
7. How do you see patients use such a personalized teaching framework?	Yes, I do see patients using this personalized teaching framework. I can see it being a helpful resource if someone was newly diagnosed to learn more. It could also be used when a clinician observes that the patient is lacking information and/or compliance in a specific area and it can be used as [a] teaching opportunity/reminder for the patient. What would the delivery methods be for this, it is [sic] solely online? Issue [sic] I can see around [sic] this: No access to computer and/or internet. Computer literacy.	To facilitate self-assessment and motivate learning in self.	Online? Please see final comments.
**Other**			
8. Are there any changes or additions to our personalized teaching framework that you would recommend?	For AF Q&A BUK # 3—I got the answer wrong and I think that most would. Based on the information given in the box, I wouldn’t have chosen to [sic] any of the possible answers. I would have probably eaten 4 asparagus spears at 48 mcg of vitamin K. Logic for this: I saw that green leaf lettuce as 45 mcg and Romaine lettuce was 48 mcg. So, of all the things on the list, the 4 spears of asparagus was the closest to that number in terms of mcg’s [sic] of vitamin K. It didn’t dawn on me to do a calculation like the answer	Tailor the framework differentially for different patient segments. If you use test subjects for multiple lessons then you’ll know how elements of the whole hang together within segments.	It is unclear how this is to be personalized. By patients [sic] level of understanding? If so, how is this determined? By drug type? e.g., Warfarin vs. Xarleto? I am unsure of the total benefit to a patient of this, with all due respect. It is difficult to assess not knowing how it is personalized, nor how the patient would access it. Would they be obliged to use it? Choose it electively? Based on my experience on Warfarin about 9 years ago, I would say there are 3 essential things. I know this is anecdotal, but for what it is worth— 1. The acclimation period presents many challenges to the patient and needs greater support than what follows. That is, until the level of the drug stabilizes in the patient. 2. Essential information is how to correct for a missed dose. The message is fairly succinct and could be communicated by the physician at the outset. 3. On warfarin, it is highly useful to know the Vitamin K content of various foods. At one time, there was an iOS app for this, and I am sure there are resources on the web as well.

**Table A3:** User-centered design: feedback received from patient representative end-users’ group for the lower back pain scenario and a low comprehension level.

Question	Patient 4	Patient 5	Patient 6
**Perceived Ease of Use**			
1. Will the purpose of the lessons be clear to patients?	Agree (4). The purpose of the lesson is clear but left me with wondering why I am not learning more about my condition and how I could at least slightly improve my situation.	Agree (4). I think it’s clear [that] the purpose of the lessons is to help patients gain knowledge to better understand what’s going on with their backs, the causes of their pain and what can be done to alleviate it. The lessons could also importantly correct wrong information or misunderstandings, and reinforce correct knowledge already obtained elsewhere (from your physio, for example). The knowledge gained could help patients understand the therapies/exercises/drugs/interventions that are being recommended by health professionals as well as put them in a better position to support strengthening and healing their backs by knowing more about what to do/what not to do and why. Some of these points about the purpose might be emphasized to make it clearer to patients how this process could help them.	Neutral (3). It is not clear what the goal is. The first question seems targeted at the patient understanding the difference between a symptom and contributing factors. There are other important symptoms such as sciatica etc. I guess my criticism is that the logical progression in this example is not clear, but I think the format is good
2. Will the structure (Q&A followed by Content BUK) of the lessons be clear to patients?	Strongly Agree (5). The structure is logical as first, it defines a condition for the patient (what) and then provides some guidance for a patient’s behavior (how).	Strongly Agree (5). The interactive structure is more interesting and relevant than googling and reading long articles. Multiple choice Q and As are more fun, frankly. Info presented in this interactive way and in shorter sections is also easier for most people to retain.	Strongly Agree (5). [My] only concern is (as you have already stated) [that] this may be too basic for some patients, making it non-interesting and less informative.
3. Will the transitions between questions and explanations be intuitive and understandable to patients?	Strongly Agree (5). The transition is appropriate, please see my explanation provided in 2).	Agree (4). I think the transitions are understandable. I’m not sure what you mean by the transitions being intuitive unless it’s that the framework is multiple choice questions and answers, so it’s obvious what the process is. I think as one keeps answering the questions it becomes apparent that the depth of information and the tailoring to one’s personal situation increase with the right answers. (If one gets a wrong answer, then is a there a more basic question next that helps with something more foundational?)	Agree (4). This can be done. The example lacks a bit of this, but the structure allows for this concept to be true.
**Perceived Usefulness**			
4. Is the content of the lessons appropriately tailored to different level of health literacy?	Agree (4). I believe that the content should be appropriate to a patient with the basic level of health literacy; I would, however, replace such words as ‘manifests’ and ‘exacerbate’ with a more common vocabulary to assure that the explanations are very clear to a patient regardless of her/his literacy level.	Strongly Agree (5). Yes, in the two questions in the sample, the first was more basic than the second and the second built on the first in a way that was more personalized to treatment. The answers also addressed the incorrect choices, which I think will also be helpful to increasing health literacy.	Neutral (3). Hard to say without more examples. This example seems very basic.
5. Can having access to personalized materials via lessons help patients to better manage their condition?	Agree (4). For the majority of patients, yes but it very much depends on individual. However, I believe that if it helps just one patient it should be developed and made available to all when possible	Strongly Agree (5). It has always been frustrating for me that the only way to obtain personalized information about my back issues is to go to a physio/chiropractor/doctor. They are obviously the first and very important line of support, but one doesn’t go to these therapists indefinitely, nor is one able to ask them questions between visits. Even at a visit one doesn’t necessarily ask the right questions or properly take in all the information presented. Back pain can be complex and not easy to understand so something educational tailored to me as an individual with a specific kind of back pain would be a very helpful complement to a program of therapy/exercise and support my efforts to manage my situation daily.	Strongly Agree (5). I really support the concept and if it can be tailored to different levels of understanding due to educational level, language barriers etc, it should be a useful tool.
6. Would you recommend such a personalized teaching framework to every patient?	Yes, please see my explanation provided in 2) above.	I know I would like such a tool and framework and likely the majority of patients would, but I’m not sure I can answer with a yes or no for everyone. It might depend on literacy levels, for example, or how much people are motivated, or how much time this will take. This latter piece [sic] around time would be important to let people know about. 10 min a day? 20? Or is it structured differently in terms of time?	Yes, but only once I am convinced that it can be tailored to different levels.
7. How do you see patients use such a personalized teaching framework?	It is a tough question because in my view there is no ‘one solution fits all’. Options could be developed based on existing behavioral models. The most appropriate one would be deployed by a physician treating the patient and should be based on the physician assessment of the patient.	This is a good question because this framework is about a learning path rather than having a specific question about one’s back pain and using a tool that lets you plug in the question and up pops the answer(s). How it’s presented to patients would be important. For me this would be about learning about my back, the origins of the pain in my particular case (Is it muscles, discs, nerves? Some of the above? All of the above?); then understanding why health care professionals are recommending certain exercises/therapies/drugs (how will those help), and what my part is in helping to ensure these are effective. Sometimes this is obvious, but at others [sic] it is not clear how to manage the pain or what is possible in terms of pain reduction or resolution. The teaching framework could also provide useful knowledge and info about how to best describe the pain and what questions to ask when I do see a health care professional.	Pre-op education. As a recent patient I would find this very helpful.
**Other**			
8. Are there any changes or additions to our personalized teaching framework that you would recommend?	I don’t have any comments	No, not really. I would just say the more personal and tailored to the individual the better, otherwise it won’t be as useful. Also, perhaps a way to continue the learning path if a new pain presents itself rather than starting over (probably you have thought of this already).	NO, other than what I have commented on above.
